# Establishment and long-term maintenance of primary intestinal epithelial cells cultured from the rainbow trout, *Oncorhynchus mykiss*

**DOI:** 10.1242/bio.032870

**Published:** 2018-03-15

**Authors:** Laura M. Langan, Stewart F. Owen, Awadhesh N. Jha

**Affiliations:** 1 School of Biological and Marine Sciences, University of Plymouth, Plymouth PL4 8AA, UK; 2 Global Sustainability, AstraZeneca, Alderley Park, Macclesfield, Cheshire, SK10 4TF, UK

**Keywords:** 3Rs, Environmental toxicology, Rainbow trout, Fish gut, In vitro model

## Abstract

A novel method for the establishment and long-term maintenance of *ex vivo* cultures from intestinal regions of the rainbow trout, *Oncorhynchus mykiss* (Walbaum), is reported. Adherence of cells was observed within hours, epithelial island formation recorded at 48 h and rapid proliferation with confluence achieved between 9-14 days. In addition to metabolic characterisation, basic morphology of growing cells was characterised using histology, immunofluorescence, transmission electron microscopy (TEM) and transepithelial electrical resistance (TEER). Regional differences in intestinal ethoxyresorufin-*O*-deethylase (EROD) and 7-ethoxycoumarin-*O*-deethylation (ECOD) activities in these primary grown enterocytes were compared following exposure to model inducers [i.e. *α*-NF, *β*-NF, B(*a*)P] which demonstrated significant differences. Regional differences in dietary uptake and metabolism of contaminants can therefore be studied in this *in vitro* system to increase our understanding of fundamental processes, while concurrently providing a means to reduce the number of fish required for biological studies in line with the principles of the 3Rs (Reduce, Refine and Replace).

This article has an associated First Person interview with the first author of the paper.

## Abstract (precis)

**Summary:** Understanding chemical uptake from the diet is difficult in live fish: we developed long-term intestinal cell cultures that enables the science and provides an alternative method.

## INTRODUCTION

The principles of the 3Rs (Reduce, Refine and Replace) (Russell and Burch, 1959) have become essential considerations in the design of scientific experiments utilising animals, which often require adherence to country-specific regulations. In the UK, all laboratory work with vertebrates is regulated through the Animal (Scientific) Procedures Act, 1986 (https://www.legislation.gov.uk/ukpga/1986/14/contents), and great emphasis is placed on work which replaces, reduces or refines the use of animals. Importantly, new and more sustainable methods, which minimise animal usage, has seen the development of novel *in vitro* methods specifically to address the use of fish (Langan et al., 2017; Baron et al., 2012; Kawano et al., 2011; Lee et al., 2009, 1993; Bols et al., 1994a). These alternative methods allow novel and fundamental scientific questions to be addressed that were not possible using *in vivo* or the whole animal system, so taking the science beyond the 3Rs. Tissue level responses can be studied using miniaturised organoids which are developed from primary isolated cells representing a more physiologically relevant model than isolated cells. To illustrate this, a primary fish gill and liver culture were developed from rainbow trout (*Oncorhynchus mykiss*; RT) in the early 1990s (Pärt et al., 1993; Flouriot et al., 1993). Recent improvements in the techniques of both the gill (Maunder et al., 2017; Schnell et al., 2016; Wood and Pärt, 1997) and the liver (Uchea et al., 2015, 2013; Baron et al., 2012) have led to the use of these systems to study pharmaceutical metabolism, as environmental monitoring systems and transport (Baron et al., 2017; Schnell et al., 2015; Stott et al., 2015). Concurrently, there has been an increase in studies which extrapolate biotransformation data from *in vitro* to *in vivo* scenarios (Cowan-Ellsberry et al., 2008; Nichols et al., 2006), allowing for the derivation of bioconcentration factors (Nichols et al., 2013). As reviewed by Sneddon et al. (2017), data on basic characteristics of chemical uptake, metabolism and excretion of organoid cultures, provide scientific rigour that supports the use of these cultures as animal alternative testing procedures for bioconcentration and toxicology studies. However, missing from these developed or developing *in vitro* fish models is the intestine. This model is necessary to elucidate chemical uptake, metabolism and excretion due to the increasing importance of dietary uptake of environmental contaminants.

Historically, chemicals with a log K*_ow_>*6 have been thought to be entirely taken up by the intestine (Heath, 1995), with later work establishing that dietary uptake via the gastrointestinal (GI) tract predominated at log K*_ow_* up to 7.5 (Qiao et al., 2000). In Europe, regulations concerning the Registration, Evaluation, Authorisation and restriction of Chemicals (REACH) have resulted in thousands of chemicals requiring further animal testing such as with OECD Test No. 305 [Bioaccumulation in Fish: Aqueous and Dietary Exposure; OECD (2012)] and the inherent difficulty of conducting such a test for hydrophobic substances of low solubility (Treu et al., 2015).

Dietary uptake studies such as the OECD 305 are expensive, time-consuming and subject to ethical and societal considerations of animal use. Thus, the development of an animal replacement model would address both regulatory requirements in addition to ethical and societal requirements. Development of intestinal alternative models (human, mammal or fish) have however been hindered by difficulties in culturing of this organ. The short life span of enterocytes may significantly impact on the viability of extracted cells [i.e. humans 3-5 days (van der Flier and Clevers, 2009); fish 16-122 days (Chikwati et al., 2013)] in addition to intestinal specific biological responses, microbial infections and complex interactions with extracellular matrices. Here, we describe a method for generating an inexpensive, organisationally and metabolically complex organotypic *in vitro* model derived from the RT intestine. We assess its metabolic complexity in terms of cytochrome P450 (CYP) enzymes using the ubiquitous contaminant benzo(*a*)pyrene [B(*a*)P] *ex vivo*, which has already been shown to be metabolised by the intestinal RTgutGC cell line (Langan et al., 2018). Given that RT is already an established model species for environmental and ecotoxicological testing, this model has the potential to act as a platform which can be developed further to study *in vitro* intestinal biology, ADME (i.e. absorption, distribution, metabolism and excretion) processes, pharmacological and/or toxicological testing in line with the tenets of the 3Rs. The data herein outline the methodology required to generate RT intestinal models and provide basic structural, physiological (i.e. electrical) and metabolic characterisation.

## RESULTS AND DISCUSSION

**Fig. 1.**
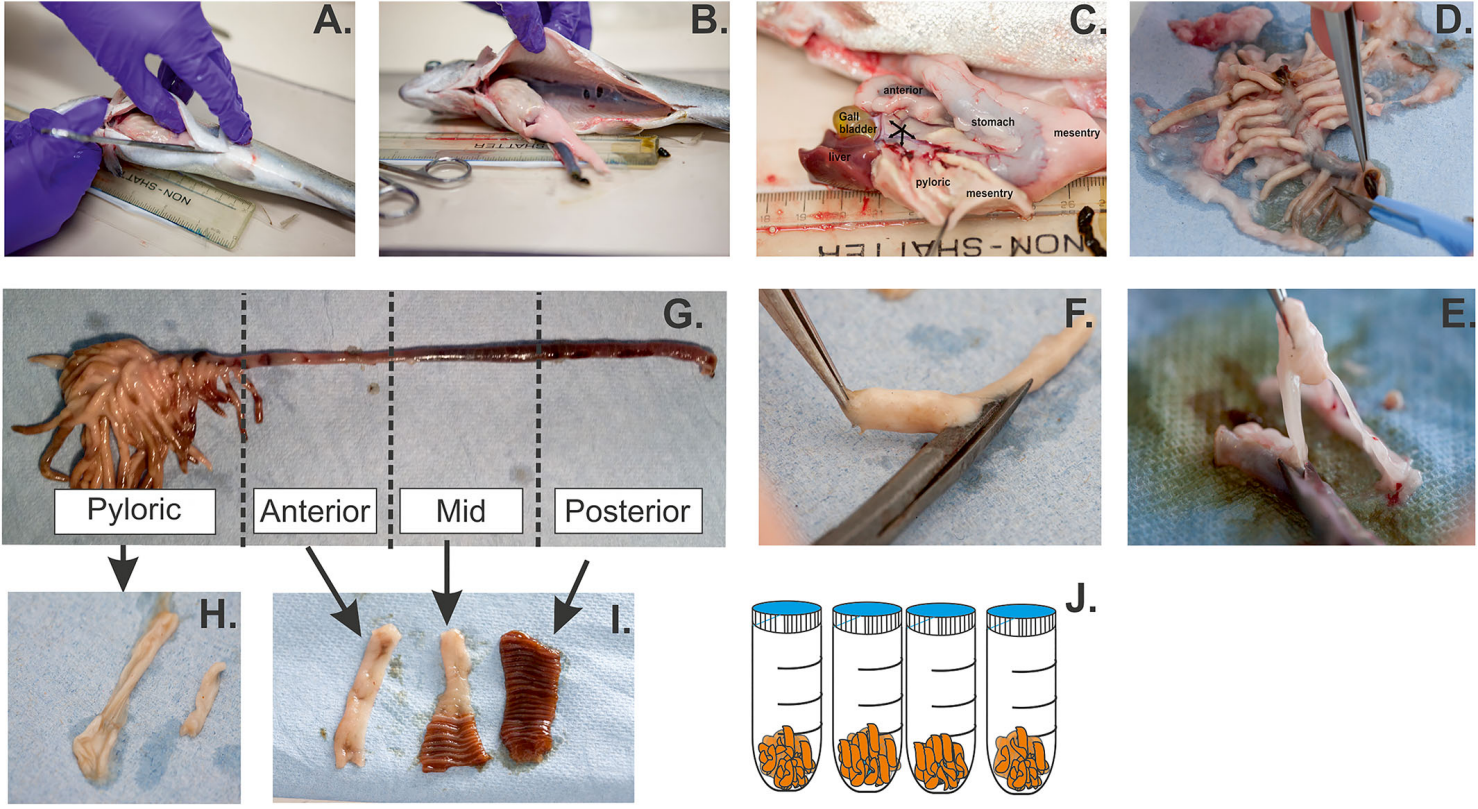
**Standardised methodology for intestine removal and preparation for cellular isolation.** (A) incision from isthmus to anus; (B) gentle removal of organs; (C) identification of structures; (D-F) removal of mesentery, fat and vasculature; (G-I) identification and separation of the regions, and finally (J) mucolytic wash and disassociation steps which occur in 50 ml collection tubes.

**Fig. 2.**
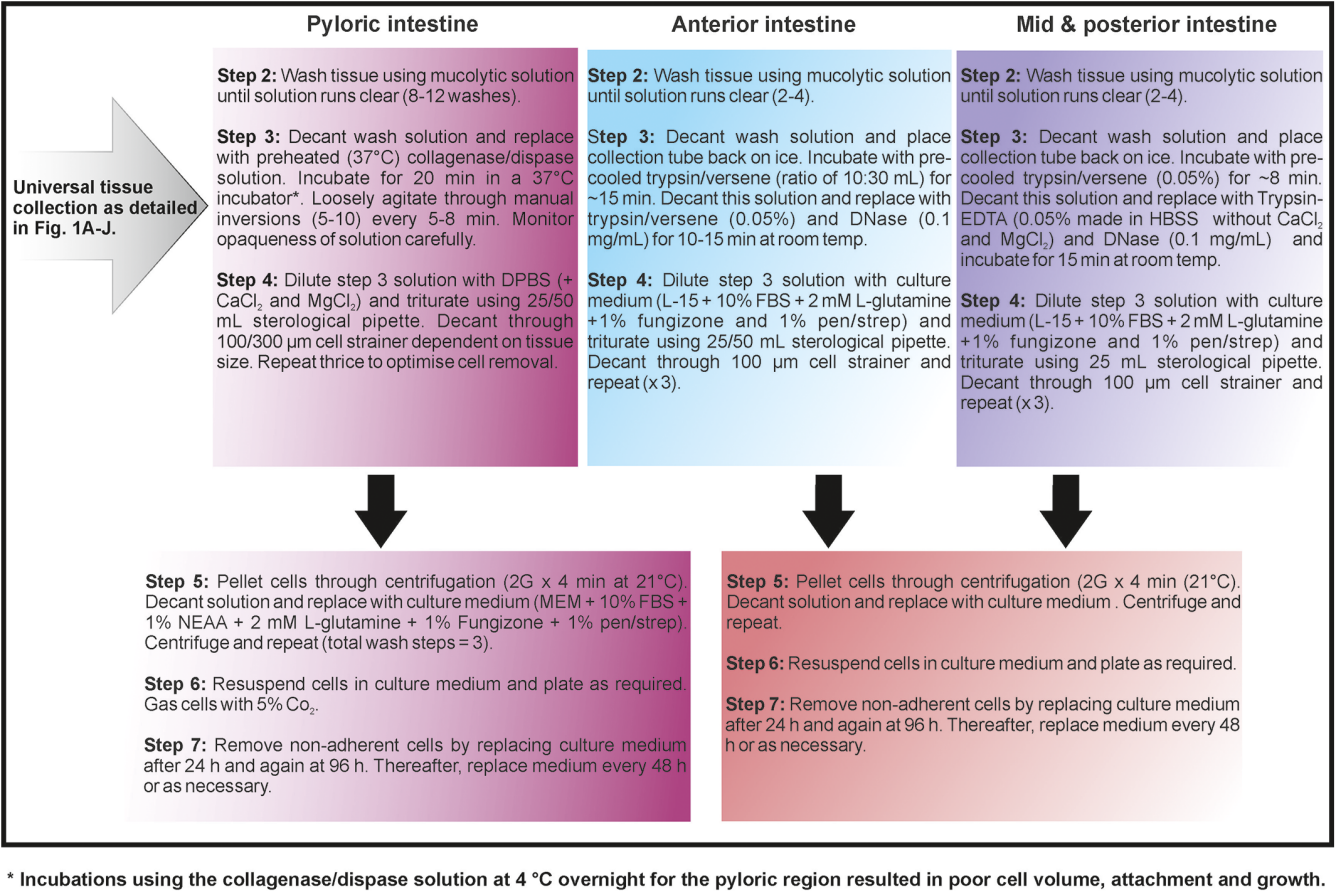
**Schematic representation of enterocyte cell isolation protocols following enzymatic treatments.** Note that this protocol explains cellular isolation following standardised methodology outlined in Fig. 1 with initial DTT treatment.

This study standardises the isolation of RT intestinal cells into three critical components: (a) standardisation of tissue preparation (Fig. 1A-J), (b) identification of intestinal region specific protocols (Fig. 2) and (c) specialist observations for successful intestinal isolation. Primary isolated cells, while more difficult to obtain than cell line maintenance, contain an enriched representation of cells more physiologically comparable to those *in vivo*, making them an important new animal alternative system. Successful and repeatable culturing of the intestinal cells starts with standardised dissection and tissue preparation outlined in Fig. 1. Thereafter, we investigated a number of methodologies outlined in detail by Kaeffer (2002) and Quaroni and Hochman (1996) due to inherent commonalities among mammalian and fish systems. However, preliminary studies using these methodologies did not result in consistent cultures. In fish systems, differences in toxicological responses between intestinal segments (Klinck and Wood, 2013; Kwong and Niyogi, 2008), cellular proliferation and differentiation (Chikwati et al., 2013) are well established.

**Fig. 3.**
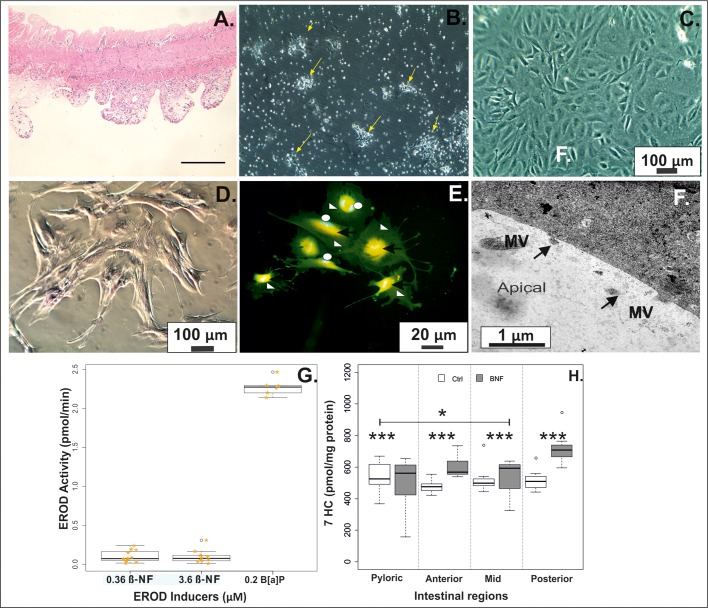
**Morphological and metabolic characterisation of cultures of primary isolated intestine cells.** (A) Tissue was collected post cellular isolation (Step 4: Fig. 2), paraffin embedded and stained with Haematoxylin and Eosin (H&E). The uppermost enterocyte layer is clearly absent, with minimal perturbation of the cellular supporting architecture. This was confirmed through (B) epithelial clusters 48 h post isolation and (C) predominance of epithelial morphology following 9 days in culture (pyloric). (D) Staining for mucosubstances demonstrates a dominance of neutral mucosubstances in the mid intestine (9 days), although this does vary between regions. (E) Immunofluorescence microscopy of pyloric cells (7 days), fixed and stained with antibodies specific to ZO-1 (red), E-cadherin (green; open triangle) and DAPI (blue; Open circles on boxplots represent data outside the interquartile range of the data) for nucleus stain. ZO-1 and E-cadherin are known as cell adhesion associated substances and when co-expressed (black arrows) may play an important role in cellular differentiation. Proteins are clearly co-expressed in cultures as can also be seen in intestinal tissue (Fig. S2). (F) TEM revealed polarised cultures with micro-villi (MV) projections (arrows) after 9 days in culture (posterior intestine), a trend observed throughout all cultures. In addition to polarised MV, cultures also maintained tight junctions, endoplasmic reticulum and lysosomes. (G) Activation of EROD activities in pyloric cultures using known inducers, with significant differences only observed with the use of B(*a*)P (*n*=10, *P<*0.001) relative to controls. This trend was uniformly observed for all intestinal cultures. Gold stars represent jittered individual data points. (H) Activation of ECOD activity was measured using a 24 h *β*-NF exposure. Significant differences were found between control and exposure in all cultures (*P<*0.001), in addition to significant differences observed between the pyloric and mid response (*P<*0.05). Box and whisker plots (G,H) display the distribution of data based on the first and third quartile and median of the data, with open circles representing data outside the interquartile range.

The current study revealed the need for region specific modifications in enzymatic concentration and duration using a combination of enzymatic solutions and mechanical disruption (Fig. 2), one of the most well-reported combinations utilised for successful cultures of numerous animal tissues (Mahe et al., 2015; Goodyear et al., 2014; Pan et al., 2012; Yoshikawa et al., 2011; Kaushik et al., 2008; Hansen et al., 2000). Our methodology results in the removal of the top layer of enterocyte cells (Fig. 3A) in a ‘sheet’-like formation thereby preserving the original cell-cell contacts. We suggest this minimises anoikosis (detachment induced apoptosis) and therefore allows for the prolonged survival of the RT isolated intestinal epithelial cells up to 6 weeks with regular media changes. Additionally, high levels of cell retrieval was observed with an average cell number for the pyloric, anterior, mid and posterior intestine of 2±0.6, 4.8±2.3, 5.0±1.3 and 5.0±4.6×10^6^ cells per tissue respectively (*n*=6 experiments) (average weight of distal intestine per region=0.35±0.13 g).

Critically, we suggest the final component of a successful isolation lies in temperature and speed. Higher concentrations of enzymes in isolation solutions in addition to longer incubation times are typically used during cellular isolation in mammalian species. However, prior studies in RT gill (Schnell et al., 2016; Stott et al., 2015; Wood et al., 2002; Pärt et al., 1993) and liver (Baron et al., 2017, 2012; Uchea et al., 2015; Blair et al., 1990) have identified optimal isolation methods at much lower levels, which was also observed in this study for the distal intestine (Fig. 2). In contrast to the other intestinal regions, successful isolation of cells from the pyloric region was obtained using a collagenase and dispase solution pre-warmed to 37°C and incubated at this temperature statically (Fig. 2). The success of this incubation is inconsistent with the biology of the RT itself and with earlier studies using fish cell lines which reported temperatures in excess of 30°C was lethal (Tong et al., 1997; Plumb and Wolf, 1971). However, enzyme activity of mammalian derived collagenase and dispase is optimal at 37°C. While this solution may typically be used at lower temperatures over longer incubation durations by some (Baron et al., 2012; Salinas et al., 2007), other studies have used this gentle approach at peak enzymatic activity (Tse et al., 2013; Langner et al., 2011) with little difference in cell viability as was observed in the current study. The current results suggest that species specific protocols are required for successful cellular isolation in addition to tissue specific modifications.

The choice of basal medium may be critical for success in culturing some regions of the RT intestinal epithelium. In the routine culture of many fish cells, basal medium which has been developed for mammalian cells can be used without modification as summarised in a review of available cell lines of marine and freshwater origin (Lakra et al., 2011). In the current study, L-15 medium was found to exhibit optimal growth of primary isolated anterior, mid and posterior intestinal cells, which is supported in the literature where the first immortalised intestinal cell line was developed from RT distal intestine (RTgutGC; Kawano et al., 2011). In contrast, primary cultures of the pyloric caeca exhibit impaired/slower growth in L-15 medium when compared to minimum essential medium (MEM) which showed markedly improved growth. MEM is well reported in the primary mammalian intestinal literature (Yamada et al., 2009; Macartney et al., 2000; Evans et al., 1992) and in the maintenance of intestinal cell lines such as Caco-2 (Masaki et al., 2006; Buesen, 2002), but has had limited used in fish cultures. Veillette and Young (2005) reported on the successful primary culture of pyloric caeca from the fish species *Oncorhynchus nerka*, suggesting a potential requirement for differing amino acids or other medium components dependent on the intestinal region being cultured. Bols et al. (1994b) observed a similar requirement for differing amino acid concentrations for the growth of fish cell lines derived from skin, liver, spleen and embryo. Recent cultures of human (Mahe et al., 2015) and murine (Psichas et al., 2017) small intestine have used Dulbecco's modified Eagle medium (DMEM) for growth. This medium is similar in formulation to MEM with additional amino acids, vitamins and proteins and provides support for the broad requirement of differing amino acid requirements dependent on intestinal region.

While some similarities exist between intestinal culture methodologies between species, it is important to note that optimal adherence and growth of RT intestinal cells in the present study was observed at 21°C, with colder temperatures resulting in impaired or slow growth. This trend was also observed in other RT primary cultures of the gill (Stott et al., 2015) and liver (Baron et al., 2012). This optimal growth temperature is not unusual in fish cell lines with a review by Lakra et al. (2011) highlighting the majority of fish cell lines are grown in temperatures in excess of 20°C. The results suggest that RT intestinal cells can differ in some of the requirements for primary or *ex vivo* growth, a finding also reported by Bols et al. (1994b) for fish cell lines.

The outlined methodology removes the top epithelial layer with minimal disturbance of the underlying mucosa (Fig. 3A) in all intestinal regions. Cells begin attaching within hours, with clumps of attaching and proliferating cells visible within 48 h (seeding density of ∼0.4×10^6^ cells/ml; Fig. 3B). Cells were able to proliferate rapidly (confluent within 7-14 days, region dependent) (Fig. 3C), however the cultures have a finite lifespan (∼6 weeks) with a predominantly epithelial cell population (Fig. 3C). In teleosts, Carrassón et al. (2006) established intestine region specific staining intensity of mucosubstances with the weakest staining for neutral mucins reported in the mid and posterior region, a finding also observed in RT (Fig. S1) and in primary grown cells (Fig. 3D). The presence of mucins (neutral) in the culture are indicative of the carry-over of a small percentage of goblet cells (Petrinec et al., 2005) but has been minimally reported in the literature despite its importance in absorption potential (Chougule et al., 2012; Yoshikawa et al., 2011). Positive histochemical staining of E-cadherin (E-cad) has been reported as confirmatory evidence of epithelial cultured *in vitro* cells (Beaulieu and Ménard, 2012; Yoshikawa et al., 2011), and was used in this study in combination with Zonula occluden-1 (ZO-1) to characterise the model. Immunohistochemical staining of all regions of the intestine (day 9 of growth) resulted in co-expression of both the epithelial marker (E-cad) and ZO-1 (Fig. 3E). These proteins are key for the assembly and functionality of the tight junction barrier with an important role in cellular differentiation, and normally present as a well-defined localisation along the perimeter of the cells. However, in this study there was no clear well-defined localisation of ZO-1 along the perimeter. This localisation is uncommon when compared to the RT intestinal cell line RTgutGC (Langan et al., 2017) or the mammalian Caco-2 intestinal cell line (Vllasaliu et al., 2014; Natoli et al., 2011), but the co-expression of these two proteins is not unusual *in vivo* with primary staining of comparable tissue revealing comparative expression (Fig. S2). Transmission electron microscopy (TEM) revealed polarised epithelium in all intestinal cultures with the presence of micro-villi projections (Fig. 3F). No significant differences were found in transepithelial electrical resistance (TEER) between intestinal cultures of individual regions or between preparations with an average of 60-90 Ωcm^2^ recorded which is similar to recordings in Atlantic salmon (80-150 Ωcm^2^; Sundell and Sundh, 2012). Investigations are continuing to improve fish cell models and the current proposed models through investigations of double seeding techniques (Langan et al., 2017; Schnell et al., 2016), application of artificial basement membranes (e.g. Drieschner et al., 2017; Vllasaliu et al., 2014), co-culture (e.g. Nollevaux et al., 2006) or through chemically induced differentiation.

Metabolic characterisation of the model was carried out using ethoxyresorufin-*O*-deethylase (EROD) and 7-ethoxycoumarin-*O*-deethylation (ECOD), which require active uptake, metabolism and excretion in order for any activity to be detected. In this study, *β*-naphthoflavone (*β*-NF) (0.36 µM) did not induce EROD activity in any of the intestinal cultures despite *α*-naphthoflavone (*α*-NF) (100 µM) being shown to inhibit activity when exposed simultaneously (*n*=10, *P<*0.001). A secondary experiment was carried out to identify a positive EROD inducer with B(*a*)P (0.2 µM) recording a significantly higher response in all cultures (Fig. 3G) (*n*=6, *P<*0.05). In agreement with prior *in vivo* studies (Lee et al., 2001), the lowest levels of EROD activity were recorded in the culture of the posterior intestine with differences observed between regions. Prior research on mammalian intestinal cell cultures have established maximum EROD induction at 48 h (Hansen et al., 2000), which was also observed in the present study. However, this CYP system is predominantly expressed in the liver over the intestine and comparisons of metabolism based on this expression alone could lead to misleading conclusions. ECOD activity is considered to represent the activities of numerous CYPs in fish (Andersson and Förlin, 1992) with Lee et al. (2001) reporting that a larger percentage of the cytochrome CYP3A form appears in the intestine that the liver of RT. Baseline ECOD activity was recorded at ∼500 pmol/mg protein with significant induction by *β*-NF (50 µM) (Fig. 3H) (*n*=8, *P<*0.001) recorded both between the controls and between intestinal regions. When ECOD activity is reported in the liver (either in culture or freshly isolated hepatocytes), a substrate of 100 µM is commonly used with baseline induction of ECOD activity varying from 87 pmol/min/mg protein in *Alepocephalus rostratus* homogenates (Ribalta and Solé, 2014) and ∼7 pmol/min/mg for freshly isolated RT hepatocytes (C. Uchea, The utility of trout hepatic cells in the prediction of xenobiotic bioaccumulation and environmental persistence, PhD thesis, University of Birmingham, 2013). The high levels of baseline ECOD induction in the current research suggest the maintenance of metabolic pathways in this model comparable to zebrafish larvae studies (∼400 pmol/larvae; Jones et al., 2010), with differences between the current model response and the liver reiterating the already established differences in metabolic response between these two organs. Following a 24 h exposure to rifampicin (a known CYP3A inducer) significant differences in ECOD activity were found between the proximal intestinal cultures (pyloric, anterior and mid intestine) and the distal posterior intestine (*P*<0.001); with the posterior intestine showing the highest activity level highlighting differences in CYP3A activity between regions. Interestingly, this has been previously reported in RT (Lee et al., 2001) and channel island catfish (James et al., 2005) and supports the maintenance of cellular process in the *ex vivo* cultures.

The primary culture of intestinal cells is a difficult process. The methodology outlined in this study demonstrates that epithelial cells isolated from all regions of the RT intestine can for the first time be maintained in culture, survive and retain comparable morphology *in vitro* to existing *in vivo* constructs. Although suggested in other organisms (Kaminsky and Zhang, 2003), it remains unknown to what extent the intestine of fish plays in first-pass metabolism of orally ingested compounds. The induction of CYPs via metabolic activation in this study provide evidence to suggest this system could be used for the examination of this substantive area of fundamental research without the extensive use of animals. Further, the opportunity for this organotypic epithelium *in vitro* model to provide both inter- and intra- individual biological replication strengthens the science beyond what is possible *in vivo*. This is a significant step for the 3Rs approach where the model can *Reduce* the total number of fish required (a dietary uptake study may use many 10's of fish), potentially *Replace* the *in vivo* studies and offer the *Refinement* that live fish are not exposed to potentially toxic chemicals. Primary cultures of the intestine will allow for tissue specific investigations into gene expression and response and will further our basic understanding of this fundamental organ. Further characterisation will provide much needed weight of evidence to fully exploit the potential of this new intestinal model and we encourage other laboratories to explore the model.

## MATERIALS AND METHODS

### Reagents

All reagents were purchased from Sigma-Aldrich (UK) or Life Technologies (UK) unless otherwise stated. Aliquots of Dithiothreitol (DTT) (Life Technologies) were pre-prepared in distilled H_2_O and stored at −20^°^C. The wash solution (mucolytic solution) used in the initial steps of enterocyte isolation contained 1% penicillin/streptomycin and an aliquot of DTT dissolved in Hanks Buffered Saline Solution (HBSS) with MgCl_2_ and CaCl_2_ which gave a final concentration of 1 mM. Fresh wash solutions were made for each isolation due to DTT's reduced activity at room temperature as previously observed by Goodyear et al. (2014). Solution formulations can vary between manufacturers and significantly impact the type and success of primary cultures of RT tissue as previously observed by Ganassin and Bols (1996) in cultures of RT spleen. As such, trypsin (25050014), versene (15040033) and trypsin/EDTA (25300054) were purchased from Life Technologies. The enzyme digestion solution contained 0.1% collagenase D (COLLD-RO, Roche) and dispase II (D4694, Sigma-Aldrich) (equivalent to 1 mg/ml) with DNase (0.1 mg/ml) and 1% FBS/BSA. This solution is pre-warmed to 37°C prior to enterocyte isolation as is routine in many primary culture protocols.

### Ethics statement

Female RT were obtained from a local supplier and held at University of Plymouth aquarium facilities. Typical husbandry conditions consisted of groups of 10-30 fish in 200 l holding tanks on a recirculating system of aerated, dechlorinated tap water, with temperature (15-16°C), pH (6.5-6.8), dissolved oxygen (96-98%) and photoperiod 12 h: 12 h. Fish were fed twice daily at 2% body weight/day. Fish were health assessed by an experienced aquaculturist and were free from morbidity. Fish were killed humanely under Schedule 1 of the Animals (Scientific Procedures) Act 1986 with a blow to the head and destruction of the brain. Fish were measured, weighed and used immediately. In the current experimental design, fish were not exposed to any test compounds, nor placed under any external stress including starvation prior to tissue collection.

### Target tissue dissection and isolation

Animals in good health and recently fed result in optimal enterocyte isolation, increased cell viability, adherence and growth of cells in monolayer. Selected animals (20±2 cm, 107±41 g) were humanely killed and sprayed with 70% ethanol before dissection. Fish were opened ventrally from isthmus to anus to isolate the intestinal tract (Fig. 1A-C). It has previously been established in the literature that the RT intestine can be divided into four regions (Fard et al., 2007; Uldal and Buchmann, 1996) due to varying responses to toxicant exposure (Klinck and Wood, 2011; Nadella et al., 2006). As such, this study divides the intestine into four distinct regions as outlined in Fig. 1G. In the case of the current study, a maximum of two fish were sampled per experiment. The isolation of enterocytes was typically carried out on average within 2 h of organ retrieval, although a maximum of 4 h has also resulted in adherent and growing cells although at a much smaller scale.

### Cell isolation and culture

Sterile/aseptic techniques were used throughout all the cell culture procedures. Enterocytes were isolated using methodology based on Evans et al. (1992) and Pan et al. (2012). Superficial fat, blood vessels and mesentery were removed from the intestinal tract using a combination of blunt scalpel blade and springbow scissors, which allow for better control during dissection and clean up, although normal scissors could also be used (Fig. 1D-F). All subsequent dissections took place on ice. Intestinal regions were isolated as outlined in Fig. 1A-G, and following longitudinal dissection, residual chyme and mucous was removed by covering the tissue specimen with dry paper towels. Following tissue dissection, each segment was placed in wash solution (described in Reagents) and stored on ice until the wash step (Fig. 1J).

Due to the large mass of this region, the pyloric segment was isolated first due to the time required for dissection and preparation (15-20 min). The mesentery and viscera were removed from the pyloric region as demonstrated in Fig. 1D until each caeca was visible (Fig. 1G). Thereafter, individual caeca were removed and cut open longitudinally. In this study, 20 caeca were removed (from each fish) including the pyloric tissue to which they were attached. However, all caeca can be removed and used, but the successful culture is dependent on the ability to cut open each caecum longitudinally. Tissue was dabbed with paper towel to remove excess mucous, cut into square pieces (∼1-3 mm^2^) and placed in wash solution on ice. The anterior, mid and posterior tissue was split from just after the last caeca extrusion into thirds equivalent to approximately 2 cm per region. The definition of these regions has previously been established in the literature (Fard et al., 2007; Uldal and Buchmann, 1996).

From this point, intestinal tissue was treated in three different ways and processed separately as outlined in Fig. 2. HBSS containing MgCl_2_ and CaCl_2_ were used predominantly in wash steps to support cell adhesion and agitation was kept to a minimum in order to prevent premature separation of the epithelial lining in order to minimise anoikosis as recommended by (Beaulieu and Ménard, 2012). Cell viability was assessed at step six of the enterocyte isolation procedure using the Trypan Blue cell exclusion assay, with average viability of cells *>*90% for all regions.

Following the method outlined in Fig. 2, isolated enterocytes can be seen to attach within hours of isolation, with 70% confluence attained 7-10 days post isolation and 100% confluence obtained within 14 days (cell seeding and culture vessel dependent). From our experience, we suggest the optimal period for enterocyte removal to be in the 1-2 h period following feeding. To ensure comparable results between experiments, the maintenance of environmental temperatures at 21°C and wash and enzymatic solutions at outlined temperatures is vital. In summary, the live fish were maintained at 16°C. The harvested tissues and wash solutions were maintained on ice during preparation and dissection, but the enzyme warmed to 37°C for the isolation incubation. Tissues were bathed at 37°C. On separation, the isolated cells were transferred to 21°C (room temperature) and cultured (see protocol Fig. 2).

### Morphological characterisation

Following isolation, intestinal cells were grown for a period of 7-9 days (confluence dependent), fixed with standard 4% formaldehyde saline for 40 min and stained with Periodic acid and Alcian blue to assess the presence of mucosubstances using protocols established previously (Langan et al., 2017). Immunohistochemical staining for tight junction formation (ZO-1) and E-cadherin (E-cad) was carried out as per Langan et al. (2017). Cells were grown on glass coverslips and processed as outlined with results extrapolated to cells grown on Transwell inserts as has been previously carried out by Gillespie et al. (2016).

The development of an intact intestinal epithelium was monitored periodically through blank-corrected measurements of TEER using an EVOM Endholm 12 culture cup, which is designed for epithelium with low TEER values (Langan et al., 2017). Intestinal cells were single seeded (0.2×10^6^ cells/ml) onto permeable polyethylene (PET) membrane inserts with 0.4 µm pores with a total surface growth area of 1.13 cm^2^ (Corning, Flintshire, UK) and maintained at 21°C, with half medium exchanges every 24-48 h. Finally, the ultra-structure of the cells grown on PET membrane inserts was characterised following methodology previously reported (Langan et al., 2017).

### Metabolic characterisation

Metabolic activity in the form of EROD activity was quantified using methodology previously established in our laboratory (Langan et al., 2018). Briefly, cells were seeded into black 96-well culture plates at a seeding density of 0.2×10^6^ cells/ml (200 µl per well) and allowed to become confluent over a 7-9 day period. Exposure was initiated through the removal of medium (150 µl) from the culture well and replacing it with 50 µl of exposure solution containing two well-known CYP1A inducers [0.36 µM *β*-NF and 0.2 µM B(*a*)P] in addition to an inhibitor (100 µM *α*-NF) and a solvent control (0.1% DMSO, final well concentration). Activity was recorded using a fluorescence plate reader (FLUOstar Omega, BMG LABTECH, Aylesbury, UK) using an excitation wavelength of 544 nm and an emission wavelength of 590 nm. EROD activity was standardised to protein content per well using the fluorescamine method (Kienzler et al., 2012) with bovine serum albumin (BSA) as standard. CYP3A metabolic activity in the primary enterocytes was assessed using the ECOD assay with the model inducers *β*-NF and rifampicin using the methodology of Christen et al. (2009) and Uchea et al. (2013). Rifampicin has been shown to increase CYP3A activity in the intestine of mammals (Monshouwer et al., 1998) with little activity in the liver (Smith and Wilson, 2010). Briefly, primary enterocytes in monolayer culture were allowed to grow for 7-9 days (∼70% confluence) and exposed to Rifampicin (0.01-1000 µg/l) for 24 h. Following exposure, the supernatant was removed and replaced with 7-ethoxycoumarin (50 µM final concentration) and incubated for 5 h. Following incubation, cells were centrifuged at 2000 **g** for 4 min at 4°C and 120 µl of the supernatant transferred to black 96-well plates pre-spiked with 80 µl of stop solution (80% acetonitrile and 20% 0.5 M TRIS base). The plate was read on a FLUOstar Omega spectrophotometer (BMG LABTECH) using an excitation wavelength of 410 nm and an emission wavelength of 530 nm. To control for unequal numbers of cells per well, cells were treated with trypsin, incubated in lysis buffer and the protein content quantified using the Micro BCA Kit (Thermo Fisher Scientific) with BSA standard curve (2 mg/ml) run in parallel on each plate.

### Statistics

Statistical analyses were performed in R (Version 3.1.3, The R Development Team, 2015). Data are given as the mean value±standard error of the mean (s.e.m.), with ‘*n*’ denoting replicates per experiment. All experiments were carried out on individual inserts/well with a minimum of three technical replicates per experiment. Due to the cellular extraction process, each seeded culture well contains a non-homogeneous cell population making them unique in terms of conventional methods for statistical analysis of cell culture experiments. As such, each well represents *n*=1. All data were first tested for normality using the Anderson-Darling Normality test (AD) in addition to examination of QQ-plots, while homogeneity of variance was examined using Levene's test, and an appropriate parametric or non-parametric test was then applied. Data were analysed using either an ANOVA with Tukey's post hoc or non-parametric Kruskal–Wallis test followed by Dunn's pairwise posthoc test with multiple pairwise comparison (two-tailed) using the ‘pgirmess’ package (https://cran.r-project.org/web/packages/pgirmess/index.html). For all statistical analyses, a value of *P<*0.05 was considered significant.

## Supplementary Material

Supplementary information

First Person interview
