# Supplementary material for: Establishment and long-term maintenance of primary intestinal epithelial cells cultured from the rainbow trout, Oncorhynchus mykiss
Source: Biol Open. 2018 Mar 15;7(3):bio032870. doi: 10.1242/bio.032870 (PMC5898270; doi:10.1242/bio.032870)
Supplement: Supplementary information [file biolopen-7-032870-s1.pdf]

© 2018. Published by The Company of Biologists Ltd | Biology Open (2018): doi:10.1242/bio.032870: Supplementary information

FIRST PERSON

First person – Laura Langan

First Person is a series of interviews with the first authors of a selection of papers published in Biology Open, helping early-career researchers promote themselves alongside their papers. Laura Langan is first author on 'Establishment and long-term maintenance of primary intestinal epithelial cells cultured from the rainbow trout, *Oncorhynchus mykiss*', published in BiO. Laura is a postdoctoral research fellow in the lab of Awadhesh Jha at the School of Biological and Marine Sciences, Plymouth, UK. Her research interests lie in the development, characterisation and improvement of aquatic-based *in vitro* animal alternatives, specifically of the intestine, with a view to improving toxicity predictions and reducing animal use in safety assessments.

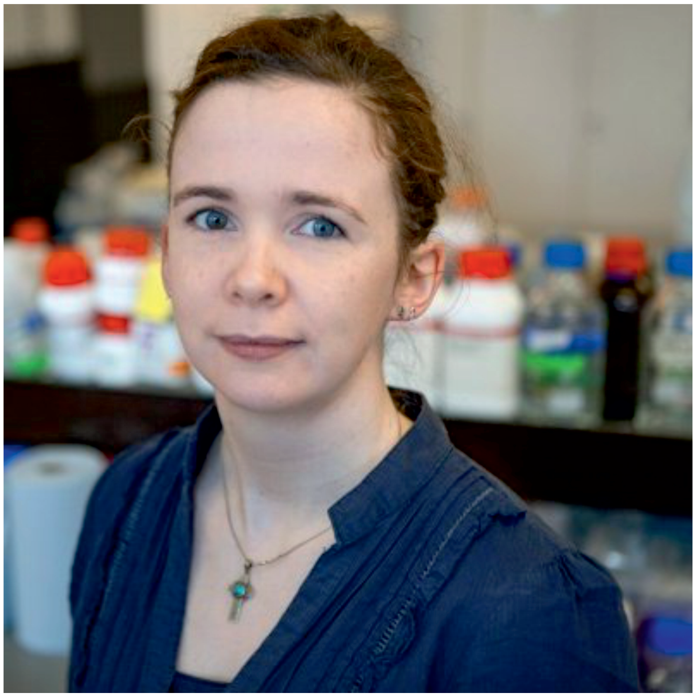

Laura Langan

What is your scientific background and the general focus of your lab?

My research background in the field of aquatic biology aims to elucidate fundamental life processes, from cellular and molecular levels up to whole animal level. My career has included working in fishery assessments of non-commercial species and in assessment of coastlines using live sampling. In this context, I realised that the sheer quantity of animals used in assessments of fisheries and in toxicity safety assessments is substantial. Population growth, industrialisation and increasing consumer demands continue to cause environmental stress in living systems. Many contaminants are finding their way into the aquatic environment, causing stress to the exposed biota, an increase in diseases, and other negative effects on wild species. The focus of the lab group where the outlined work was performed is in the broad discipline of ecotoxicology. Under this umbrella, a subset of the group worked on the development of specialised *in vitro* cultures from live cells that mimic the response of tissues in whole fish. With the development of novel cultures of organs such as the liver, gill and intestine, a 'virtual fish' alternative could be created and utilised to reduce and/or replace significant numbers of animals used in safety assessments to meet ethical and regulatory requirements, with significant economic implications.

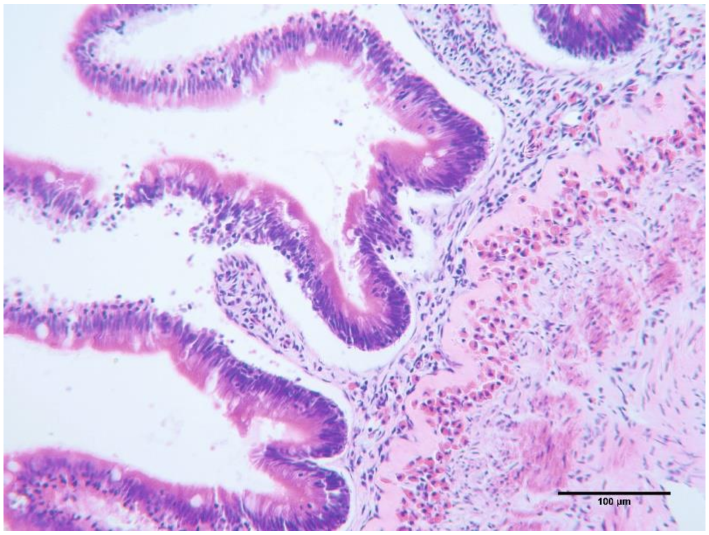

Clear selection of enterocyte cells in isolation procedure of rainbow trout intestine using short incubation time.

How would you explain the main findings of your paper to non-scientific family and friends?

All chemicals essential for modern life need to be safe for us and the environment. Before any chemical product can be used or consumed by humans or discharged in the environment, it must first undergo safety assessments. Tens of thousands of chemicals are awaiting these types of assessments, but these take time, are costly and may use hundreds of animals per chemical compound. My work looks at ways we can *reduce*, *refine* or completely *replace* the animals used in these assessments. This is called the 3Rs and is a commitment to better science, an important vision for hazard and risk assessments of chemicals. Within the area of these assessments, dietary uptake of compounds (taken in through the food chain or via ingestion) is poorly understood. My research establishes a way to increase our understanding of dietary uptake in fish, an important group of organisms used for regulatory testing, without using substantial quantities of animals. This approach also aims to minimise our personal exposure to hazardous compounds that may accumulate in fish tissue. Overall, my work is aimed at helping to protect the human and natural environment.

Laura Langan's contact details: School of Biological and Marine Sciences, Drake Circus, Plymouth, PL4 8AA, UK.

E-mail: laura.langan@plymouth.ac.uk

Biology Open (2018): doi:10.1242/bio.032870: Supplementary information

What are the potential implications of these results for your field of research?

The information and methodology in this study provides a functionally comparable animal replacement model that provides both inter- and intra-individual biological replication and strengthens the science beyond what is possible *in vivo*. The work represents a significant step for the 3Rs approach where the proposed models can *reduce* the total number of fish required, potentially *replace* the *in vivo* studies and offer *refinement* that live fish are not exposed to potentially toxic chemicals. Importantly, primary cultures of the intestine will allow for the further exploration of the emerging areas of *relevance* and *read-across*, potentially allowing for reductions in mammalian and human testing.

"I love the challenge of science, the continuous learning, problem solving and divergent paths."

What changes do you think could improve the professional lives of early-career scientists?

The first thing that comes to mind is funding opportunities, but there needs to be a fundamental change to the traditional postdoctoral system to provide a stable work/life balance. Early-career scientists typically work all hours possible to get those coveted publications, with the permanent fable that more publications will eventually lead to job security over the years, moving the science forward and eventually establishing the field. In this context, an interesting idea being trialled at the Francis Crick Institute in London is to give early-career researchers a new and improved contract. Complaints about short-term contracts, incessant grant applications and a lack of independence were listened to and new employment contracts (initially 6 years to a maximum of 12 years) were developed. The institute calls it a 'family friendly' approach, but importantly, researchers can focus on the science and their personal development. Currently, teaching universities do not offer stable support like this, and short-term contracts are the only option with a shrinking source of funding. A change to research contracts at universities could offer a significant improvement to the professional lives of early-career researchers.

What's next for you?

Since completing my PhD, I have started a postdoctoral fellowship at the University of Plymouth. While working on the new project, I have continued to complete some of the tasks for my previous project, expanding my research to incorporate both gill and liver metabolism in rainbow trout.

I love the challenge of science, the continuous learning, problem solving and divergent paths. When I started my career, all I thought about was building a functional intestinal model to reduce animal use in assessments. Currently, I am challenging myself to take on omics-based approaches to characterising xenobiotic organs in the biota, and how this could impact our fundamental understanding of chemical–biological interactions. I am continuously building on my toolbox of techniques and understanding. These skills support my scientific mind-set to develop a career where I can adapt to new challenges and help develop solutions to help society and improve the quality of both human life and the environment.

Reference

Langan, L. M., Owen, S. F. and Jha, A. N. (2018). Establishment and long-term maintenance of primary intestinal epithelial cells cultured from the rainbow trout, *Oncorhynchus mykiss*. *Biol. Open* **7:** bio032870.

Biology Open
